# Adenovirus based virus-like-vaccines targeting endogenous retroviruses can eliminate growing colorectal cancers in mice

**DOI:** 10.18632/oncotarget.26680

**Published:** 2019-02-15

**Authors:** Lasse Neukirch, Tea Kirkegaard Nielsen, Henriette Laursen, Joana Daradoumis, Christian Thirion, Peter Johannes Holst

**Affiliations:** ^1^ University of Copenhagen, Center for Medical Parasitology, The Panum Institute, DK2200 Copenhagen, Denmark; ^2^ Clinical Cooperation Unit Applied Tumor Immunity, National Center for Tumor Diseases and German Cancer Research Center, 69120 Heidelberg, Germany; ^3^ Sirion Biotech GmbH, 82152 Planegg-Martinsried, Germany; ^4^ InProTher ApS, DK2200 Copenhagen, Denmark

**Keywords:** adenoviral vectors, cancer, endogenous retroviruses, virus-like particles, virus-like-vaccines

## Abstract

Endogenous retroviruses (ERVs) that make up 8% of the human genome have been associated with the development and progression of cancer. The murine model system of the melanoma associated retrovirus (MelARV), which is expressed in different murine cancer cell lines, can be used to study mechanisms and therapeutic approaches against ERVs in cancer. We designed a vaccine strategy (Ad5-MelARV) of adenoviruses encoding the MelARV proteins Gag and Env that assemble *in vivo* into virus-like particles displaying the cancer-associated MelARV Env to the immune system. The novel vaccine was designed to induce both humoral as well as cellular immune responses in order to attack ERV expressing tumor cells. Despite a lack of antibody induction, we found that T cell responses were strong enough to prevent colorectal CT26 tumor growth and progression in BALB/c mice after a single vaccination before or after tumor challenge. A combination with the checkpoint inhibitor anti-PD-1 further increased the efficacy of the vaccination leading to complete tumor regression. Furthermore, immune responses in vaccinated mice were not restricted to only one cancer cell line but vaccinated animals were also protected from a rechallenge with the distinct breast cancer cell line 4T1. Thus, the developed vaccine strategy could represent a novel tool to successfully target diverse ERV-bearing tumors in cancer patients.

## INTRODUCTION

Endogenous retroviruses (ERVs) are the evidence of ancient retrovirus infections in our distant ancestors. Diverse genomic integrations of viral DNA accumulated in the course of time and today around 8% of the human genome is considered to be endogenous retroviral DNA [1]. Even though the integration events occurred millions of years ago and mutations, insertions and deletions caused the ERVs to be merely relics of their former viruses, some functional ERV proteins remained and still play important roles in the human physiology and pathology [2–5]. In this context, several studies have highlighted a connection between ERV expression and the development and progression of cancer [2, 4, 6–8].

A prominent example of a human ERV (HERV) is the HERV type K (HERV-K) that is associated with prostate cancer, breast cancer, ovarian cancer, lymphomas, melanomas, leukemia and sarcomas [2, 4]. Just recently pro-oncogenic properties have been assigned to HERV-K [8] which adds to the previously discovered immunosuppressive ability [9]. As HERV-K is one of the most promising target candidates for an immunotherapeutic approach, diverse strategies have been tested already in humanized or immunosuppressed mouse models with varying success rates [10–13].

However, not only human tumors show elevated levels of ERV proteins, but also murine cancer cell lines have been found to express ERVs [14, 15]. This provides a perfect model organism to study general effects of ERVs on tumor progression and to test ERV-targeting therapies. A commonly used ERV model is the melanoma associated retrovirus (MelARV) which originates from a provirus of the murine leukemia virus (MuLV) that is integrated into the murine genome [15]. Most inbred mouse strains contain one or two inactive MuLV copies [15] that do not show expression in healthy tissues but are clearly detectable in diverse tumor cell lines [16]. Even though it is not always possible to determine if ERV protein expression is a cause or a consequence of the developing tumor, the observation that cancer cells maintain activation of these proteins, in spite of selection pressure, indicates a beneficial effect of ERVs for the tumor itself [17]. One example for an ERV gene encoded protein with pro-cancerous properties is the Envelope protein (Env) of MelARV. Originally, Env was displayed on the surface of viral particles where it was required for the binding and fusion with cell membranes [18]. The MuLV Env (and hence the MelARV Env) consists of two subunits, p15E and gp70, that are connected by disulfide bonds [19]. Similar to other retroviral Envs [20], the transmembrane subunit p15E anchors the protein in the cell or virus membrane, while the surface subunit gp70 binds to the target cell prior to fusion. Additionally, gp70 covers and shields conserved regions within the p15E subunit, such as the immunosuppressive domain (ISD) that contains the immunosuppressive peptide CKS-17 [21, 22]. This particular domain has been shown to play a role in the promotion of cancer by suppressing anti-tumor immune responses [23]. Thus, the immunosuppressive characteristic may explain why expression of MelARV Env is critical for the growth of tumors as observed by Mangeney *et al*. [24].

In the attempt to follow up with past studies [11, 25–31] we tried to target MelARV Env with an immunotherapeutic approach. Our vaccine strategy includes the presentation of MelARV Env to the immune system on virus-like particles (VLPs) which are encoded by a recombinant adenovirus type 5 (Ad5). This strategy of adenovirus based virus-like-vaccines [32] has been tested before with the purpose of targeting human immunodeficiency virus Env [33–35] and a placental malaria-associated antigen [36]. The rationale behind the vaccine was to present the ERV target protein MelARV Env on the VLPs in a rather natural, virus-like conformation to induce optimal immune responses by mimicking a real infection [32]. Therefore, we hypothesized that by displaying MelARV Env on adenovirus-encoded VLPs we would be able to induce both strong antibody as well as T cell responses against the target protein on cancer cells to prevent tumor progression. Here we show that even though the vaccine-induced immune responses were deficient in generating antibodies, elevated levels of activated T cells were able to prevent post-vaccine growth of murine colorectal cancer cells, CT26. In addition, the vaccine served as a therapeutic-like tumor protection and was not restricted to only one cancer cell line but also showed long-term protection against the distinct breast cancer cell line 4T1. Finally, in combination with anti-PD-1 treatment, tumors were eradicated with a 100% efficacy in the murine model system.

## RESULTS

### Characterization of the adenoviral vaccine vector Ad5-MelARV

**Figure 1:**
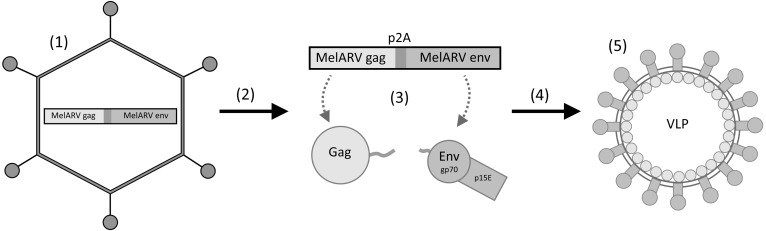
Rationale of the vaccine virus Ad5-MelARV (1) An adenovirus type 5 (Ad5) encodes the MelARV genes *gag* and *env* coupled via a self-cleavable peptide (P2A). (2) Upon injection into mice, the virus transduces target cells (3) leading to the protein expression of Gag and Env. (4) Gag proteins assemble at the cell membrane and form virus-like particles (VLPs) that integrate Env into their lipid bilayer. (5) The released VLPs present Env, consisting of the two subunits gp70 and p15E, on their surface to the immune system.

The concept of our vaccine was to co-encode MelARV group-specific antigen (Gag) and MelARV Env, coupled via the self-cleavable peptide P2A to ensure equimolar expression of both proteins, in a replication-deficient Ad5 vector (Ad5-MelARV) [32, 37]. While Gag was intended to induce formation of VLPs in vaccine vector transduced cells, Env was included as the target antigen, expressed in cancer cells, and to be displayed on *in vivo* produced VLPs (Figure 1).

**Figure 2:**
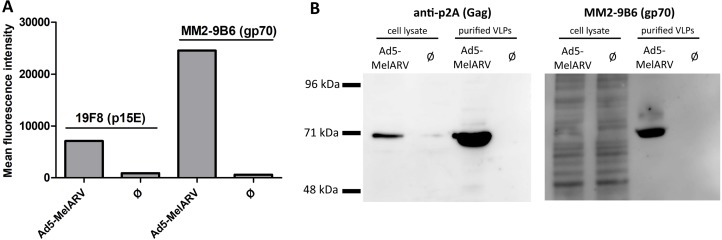
Assembly and release of VLPs by Ad5-MelARV transduced cells Vero cells were incubated with Ad5-MelARV and analyzed for expression of the MelARV Env subunits p15E (19F8) and gp70 (MM2-9B6) or MelARV Gag (anti-P2A). Cells infected with an irrelevant adenovirus served as negative controls (Ø). (**A**) Expression of the target protein MelARV Env was shown on the surface of adenovirus transduced target cells. Vero cells cultured in the presence of Ad5-MelARV were incubated with primary antibodies against MelARV Env (19F8 or MM2-9B6) and bound antibodies were detected by flow cytometry using fluorescent-conjugated secondary antibodies. (**B**) Expression of the target protein MelARV Env was shown in transduced cells and in released VLP. Cell lysates of transduced Vero cells and VLPs purified from the cell culture supernatant were analyzed by Western blot for the expression of MelARV Gag (anti-P2A) (left) and the MelARV Env surface subunit gp70 (MM2-9B6) (right).

To confirm the viral vector's ability to release functional VLPs, Vero cells were incubated with the recombinant adenovirus Ad5-MelARV. Expression of Env on the surface of transduced cells was analyzed by flow cytometry (Figure 2A), while cell lysates and released VLPs were analyzed by Western blot to confirm the presence of the encoded proteins, Env and Gag (Figure 2B).

The two subunits of Env, the transmembrane subunit p15E and the surface subunit gp70, were present on the surface of transduced cells as shown by binding of the monoclonal antibodies 19F8 [25] and MM2-9B6 [15], respectively (Figure 2A). On the contrary, cells transduced with an irrelevant recombinant Ad5 did not stain with any of the Env-specific antibodies.

Additionally, Western blot analysis of lysates and purified VLPs from Ad5-MelARV transduced cells confirmed Gag and Env expression in the cells and successful release of Env containing VLPs (Figure 2B). Lysates and supernatants from Vero cells transduced with an irrelevant Ad5 vector were employed as controls. To confirm expression of MelARV Gag, an antibody specific for the self-cleavable P2A peptide was used. The P2A peptide is encoded between Gag and Env to assure separation after translation. The larger part of the cleaved peptide remains bound to Gag allowing detection of this protein with a P2A-specific antibody. The detected band in the cell lysate and purified VLPs of approximately 70 kDa represents the MelARV Gag protein (~65 kDa [38]) plus the residual P2A contributing with about 2 kDa and eventual post-translational modifications (Figure 2B left). Expression in transduced cells and VLP incorporation of Env were confirmed by binding of MM2-9B6, an antibody detecting the MelARV Env surface subunit gp70 (Figure 2B right) [15].

Additionally, expression of the MelARV antigen from the DNA vector encoding the same construct as Ad5-MelARV was shown by Western blot through detection of Gag-bound P2A in the lysate of transduced cells (Supplementary Figure 1).

### Vaccine induced antibody responses

BALB/c mice were either vaccinated with Ad5-MelARV, DNA-MelARV (a plasmid containing the same expression cassette as Ad5-MelARV), or with both vaccines in a DNA-Ad5 prime-boost. Vaccine-induced target antibodies in the blood serum were characterized by their ability to bind MelARV Env expressing tumor cells in general and the MelARV Env transmembrane subunit p15E in particular.

**Figure 3:**
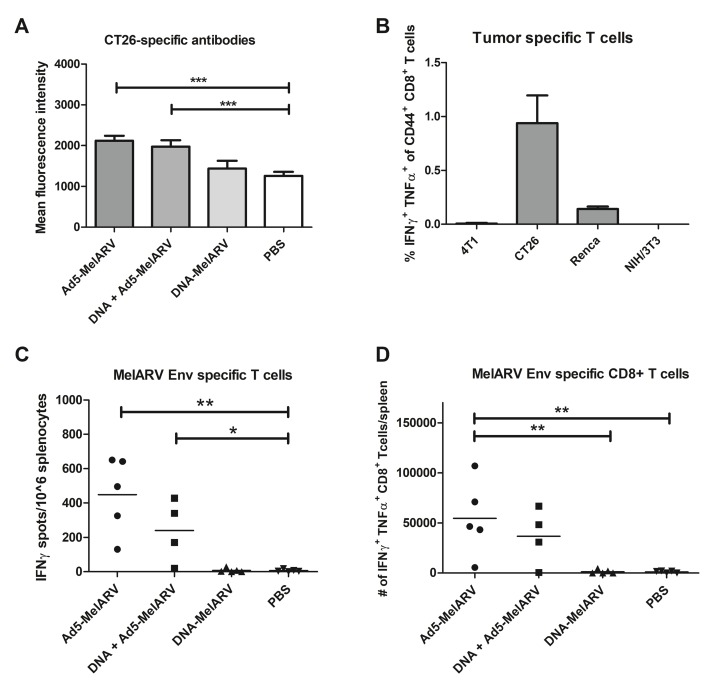
Antibody and CD8^+^ T cell specific immune responses induced by Ad5-MelARV (**A**) Analysis of tumor-specific antibodies. MelARV Env expressing CT26 cells were incubated with blood serum of Ad5-MelARV/DNA-MelARV vaccinated mice to analyze the level of tumor-binding antibodies. Bound antibodies were detected by flow cytometry using an APC-coupled secondary antibody against murine IgG. Bars show the mean fluorescence intensity of each group including the standard error of mean (SEM) with *n* = 7 (Ad5-MelARV, DNA-MelARV), *n* = 8 (DNA + Ad5-MelARV) or *n* = 9 (PBS) mice per group. Asterisks indicate significant difference to the PBS control, with ^***^(*P* ≤ 0.001). (**B**) FACS analysis of IFNγ and TNFα expression in CD8^+^ T cells stimulated with different tumor cells. Splenocytes of vaccinated mice were stimulated by co-culturing with 4T1, CT26, Renca or NIH/3T3 cells. IFNγ and TNFα expression in CD8^+^ T cells was analyzed by intracellular staining and flow cytometric analysis. Splenocytes from 5 mice were pooled and analyzed in duplicates. Bars show the percentage of IFNγ and TNFα cells in the population of CD44^+^ CD8^+^ T cells including the standard error of mean (SEM). (**C**) ELISPOT analysis of T cell responses induced by Ad5-MelARV. Splenocytes of vaccinated mice were stimulated with a peptide of the MelARV Env H2-Ld-restricted T cell epitope AH1 and activated immune cells were detected by IFNγ production in an ELISPOT assay. The result was calculated as the number of spots (IFNγ-producing cells) per 10^6^ splenocytes. Groups include *n* = 4 (DNA + Ad5-MelARV) and *n* = 5 (Ad5-MelARV, DNA-MelARV, PBS) animals. The horizontal lines represent the mean number of spots in each group. Asterisks indicate significant difference to the PBS control, with ^*^(*P* ≤ 0.05) and ^**^(*P* ≤ 0.01). (**D**) The same AH1-stimulated splenocytes as in (C) were analyzed for the expression of both IFNγ and TNFα in CD8^+^ T cells by intracellular staining and FACS analysis. The results show the total number of IFNγ^+^ TNFα^+^ CD8^+^ T cells in the whole spleen of each mouse. The horizontal lines represent the mean number of activated cells in each group. Asterisks indicate significant difference with ^**^(*P* ≤ 0.01).

Tumor-specific antibodies in vaccinated mice were analyzed by flow cytometry of the colon cancer cell line CT26 incubated with murine blood serum (Figure 3A). Mice vaccinated with Ad5-MelARV showed increased levels of CT26-specific antibodies compared to mice injected with DNA-MelARV or phosphate buffered saline (PBS), regardless if Ad5-MelARV was administered in combination with DNA-MelARV or as a single treatment. However, the observed antibody levels at the time of tumor challenge (4 weeks after Ad5-MelARV vaccination) were only increased by about 50% in comparison to the PBS control. In contrast to Ad5-MelARV, vaccination with DNA-MelARV alone had no observable effect on the production of tumor-binding antibodies (Figure 3A).

To further characterize the antibody responses, binding of serum antibodies to a specific peptide of the MelARV Env transmembrane subunit p15E were analyzed by enzyme-linked immunosorbent assay (ELISA). The chosen target peptide was a sequence proximal to the transmembrane domain that is supposedly not shielded by the surface subunit gp70 and thus relatively easy to target by vaccination [21]. However, the experiment did not show any antibody specificity for this peptide in Ad5-MelARV vaccinated mice compared to the PBS control, while a positive control serum showed specific binding to this peptide and confirmed functionality of the assay (data not shown).

### Vaccine induced T cell responses

In order to determine general tumor-specific immune responses induced by the vaccine as part of the cell-mediated immunity, splenocytes from Ad5-MelARV injected mice were co-cultured with the tumor cell lines 4T1, CT26 and Renca as well as the fibroblast cell line NIH/3T3 known to be negative for MuLV/MelARV Env [16]. While no responses where observed in 4T1 stimulated splenocytes and the negative control NIH/3T3, a weak response was observed for Renca cells. CT26 stimulated splenocytes showed the highest percentage of interferon gamma (IFNγ) and tumor necrosis factor alpha (TNFα)-producing CD8^+^ T cells (Figure 3B).

For a more specific analysis of T cell responses in vaccinated mice, splenocytes were stimulated with AH1, which is a known H2-Ld-restricted T cell epitope in BALB/c mice located in the MelARV Env surface subunit gp70 [14]. Analysis by enzyme-linked immunospot assay (ELISpot) revealed approximately 450 activated IFNγ-producing T cells per 10^6^ splenocytes in Ad5-MelARV vaccinated mice (Figure 3C). In contrast, PBS control mice showed only 6 activated T cells per 10^6^ splenocytes on average (Figure 3C). Vaccination with DNA-MelARV alone did not promote activation and expansion of AH1-specific T cells. The ELISpot results were in accordance with data obtained from a flow cytometric analysis of IFNγ and TNFα production in AH1-stimulated splenocytes (Figure 3D). Ad5-MelARV vaccinated mice showed significantly increased levels of activated AH1-specific CD8^+^ T cells compared to DNA-MelARV and PBS injected mice.

### Prophylactic vaccination with Ad5-MelARV protected mice from CT26 tumor growth

**Figure 4:**
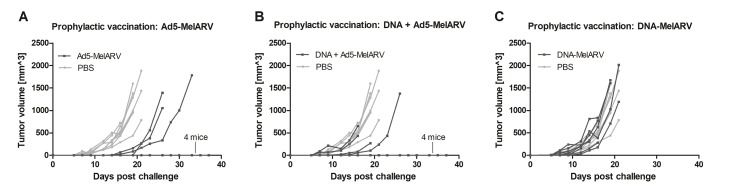
Tumor protection in prophylactically vaccinated mice Vaccinated BALB/c mice (dark lines) were challenged s.c. with 5 × 10^5^ CT26 cells and tumor growth was assessed every 2–3 days. PBS injected mice served as negative controls (light grey lines) (*n* = 8). Each line represents a single mouse in the course of the experiment. (**A**) Mice were vaccinated with Ad5-MelARV only (*n* = 7), (**B**) with DNA-MelARV and Ad5-MelARV in a prime-boost regimen (*n* = 8), or (**C**) with DNA-MelARV only (*n* = 7).

In order to test our vaccine as tumor-protecting, vaccinated BALB/c mice were challenged subcutaneously with CT26 tumor cells. While all PBS injected mice developed tumors and had to be sacrificed within 21 days after the challenge (Figure 4A–4C light grey lines), 4 out of 7 Ad5-MelARV vaccinated mice did not develop tumors at all. Additionally, all the tumor-bearing mice had delayed appearance of tumors growing as in PBS injected mice (Figure 4A). Vaccination with DNA-MelARV, on the other hand, had no beneficial effect on the protection from tumor development (Figure 4C), which is in accordance with the previously observed antibody and T cell data (Figure 3). With 4 out of 8 tumor-free mice, the combination of DNA-MelARV and Ad5-MelARV in a prime-boost regimen did not improve the protection of Ad5-MelARV alone (Figure 4B).

### Vaccination with Ad5-MelARV eradicated growing CT26 tumors

**Figure 5:**
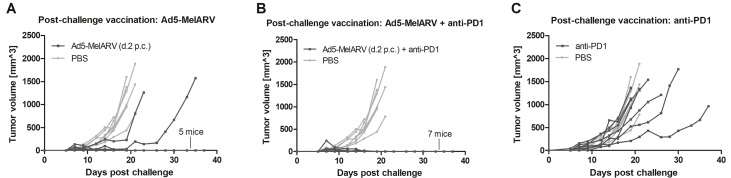
Tumor protection in post-challenge vaccinated mice Mice were challenged with CT26 cells and subsequently received vaccinations with Ad5-MelARV. PBS injected mice served as negative controls (light grey lines) (*n* = 8). Each line represents a single mouse in the course of the experiment. (**A**) Mice were vaccinated on day 2 post challenge (d.2 p.c.) with a single dose of Ad5-MelARV (*n* = 7). (**B**) Additionally to the vaccination on d.2 p.c., mice received an anti-PD-1 treatment starting on day 8 after the challenge and repeated anti-PD-1 injections on days 12, 16 and 20 (*n* = 7). (**C**) As a negative control, mice received only anti-PD-1 treatment (*n* = 7).

In addition to the previously described prophylactic vaccination, Ad5-MelARV was further tested in a setting where mice received the vaccination after challenge, when tumors were already growing. When mice were vaccinated two days after the challenge with CT26 cells, only 2 out of the 7 vaccinated mice developed slow growing tumors and had to be euthanized (Figure 5A). While the surviving mice also developed small tumors of about 50 mm^3^ shortly after challenge, these tumors subsequently decreased in size and were finally eradicated completely. Considering the fraction of surviving mice, the post-challenge vaccination (5/7 surviving mice) showed even a slight improvement compared to the prophylactic vaccination (4/7 surviving mice) shown in Figure 4. Additionally, the observed tumor protection was further improved when the administered Ad5-MelARV was combined with a treatment of anti-PD-1 antibodies (administered on days 8, 12, 16 and 20 after CT26 challenge). Similar to Ad5-MelARV alone, all mice developed initial tumors of about 50 mm^3^ which were subsequently eradicated completely due to the combinational treatment, showing a 100% efficacy (Figure 5B). In contrast, administration of anti-PD-1 alone had no distinct effect on the growth of CT26 tumors in mice as compared to the PBS control (Figure 5C).

### Protection of Ad5-MelARV vaccinated mice is primarily mediated by CD8^+^ T cells

**Figure 6:**
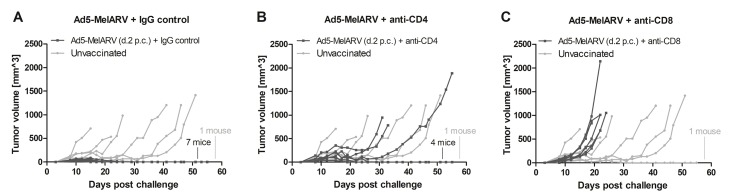
Role of CD4^+^ and CD8^+^ T cells in tumor protection BALB/c mice were challenged with CT26 cells and were vaccinated with Ad5-MelARV after two days (d.2 p.c.) (dark lines). T cell depletion antibodies were administered on days 5, 8 and 11. Unvaccinated mice served as negative controls (light grey lines) (*n* = 7). Each line represents a single mouse in the course of the experiment. (**A**) A control group of vaccinated mice was injected i.p. with rat IgG2b isotype control (*n* = 7). (**B**) Depletion of CD4^+^ T cells by i.p. injection of anti-mouse CD4 antibodies (*n* = 7). (**C**) Depletion of CD8^+^ T cells by i.p. injection of anti-mouse CD8 antibodies (*n* = 7).

In order to further analyze the mechanistic effect of the tumor protection, CT26 challenged mice were vaccinated and additionally injected with CD4^+^ or CD8^+^ depleting antibodies (Figure 6). Successful depletion of the respective T cells was confirmed by flow cytometry of peripheral blood mononuclear cells (PBMCs) (data not shown). As observed in the previous experiment, Ad5-MelARV, administered two days after the tumor challenge, led to the eradication of growing CT26 tumors when an IgG isotype control was injected (Figure 6A), although in this case all of the 7 treated mice were protected and about half the PBS injected mice showed delayed tumor growth. The administration of anti-CD4 antibodies had a moderate effect on the vaccination apparent from a slightly larger initial tumor formation and with 3 out of 7 mice that were no longer protected and developed slow growing tumors (Figure 6B). The effect was more severe after anti-CD8 antibody injection (Figure 6C). Here, the protective effect of the vaccine was completely abrogated and all of the mice developed fast growing tumors.

### Ad5-MelARV vaccinated mice were protected from tumor growth when rechallenged with the heterologous tumor model 4T1

**Figure 7:**
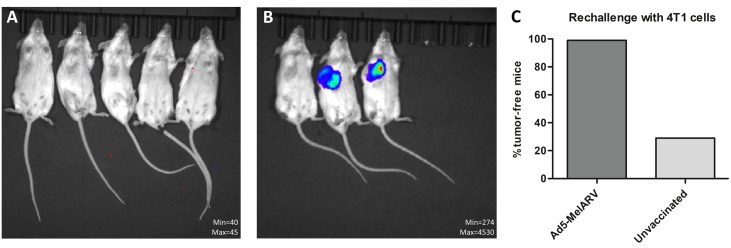
Rechallenge of vaccinated mice with 4T1 tumor cells Ad5-MelARV vaccinated mice that survived a CT26 tumor challenge (Figures 4 and 5) were rechallenged with 4T1 tumor cells injected in the thoracic mammary fat pad. *In vivo* growth of the luciferase transfected 4T1 cells was analyzed by IVIS imaging to detect tumor bearing mice. (**A**) Representative IVIS image of vaccinated mice that survived CT26 challenge and were rechallenged with 4T1 cells. (**B**) Representative IVIS image of unvaccinated, 4T1-challenged mice (negative control). (**C**) Comparison of vaccinated (Ad5-MelARV) (*n* = 20) and unvaccinated (*n* = 7) groups. The graph shows the percentage of tumor-free mice in each group.

To analyze long-term protection and to test the efficacy of Ad5-MelARV against other types of cancer, BALB/c mice that survived a previous tumor challenge with CT26 cells (Figures 4, 5) were rechallenged with 4T1 tumor cells 8 weeks after the initial challenge. Upon injection of 4T1 cells in the thoracic mammary fat pad, none of the 20 vaccinated mice showed 4T1 tumor growth (Figure 7C) as analyzed by IVIS imaging (Figure 7A). On the contrary, 5 out of 7 unvaccinated control mice developed tumors at the 4T1 cell injection site (Figure 7B, 7C).

## DISCUSSION

When treating cancer by immunotherapy, antibody as well as T cell responses are of utmost importance for the immune system to detect and kill malignant cells. Previous studies used monoclonal antibodies [25–28] or adoptively transferred T cells [11] specific for MelARV Env or the closely related MuLV Env in order to prevent tumor growth. A more practical approach that has been tested before was the vaccination with MelARV Env antigens to induce specific immune responses *in vivo* [29–31]. Even though these studies showed that MelARV Env is a relevant target protein present on different types of cancer [14–16], none of the past strategies targeting this antigen was sufficient in eradicating established tumors.

Since a general aim of a cancer vaccination strategy is to achieve broad targeting of different types of cancer, we used the MelARV Env target protein sequence, originally found in melanoma cells (e.g. B16 cells), to vaccinate against the colon cancer cell line CT26. Like several other murine cancer cell lines, both the B16F10 as well as the CT26 cells express closely related ERV Env proteins derived from the MuLV [14–16]. MelARV represents a unique B cell epitope in some melanoma cells, detectable by the monoclonal antibody MM2-9B6 [15], and differs solely in a few single amino acids from ERVs of other mice strains and cell lines. These point-mutations do not affect essential T-cell epitopes like the tested AH-1. Thus, a functional vaccination strategy with the MelARV Env target sequence could in principle be applied to many different murine cancer types, considering the very broad expression of MuLV Env-derived gene products in diverse tumor cell lines [16].

The vaccine approach tested in this study, using an adenoviral vector encoding for MelARV Env displaying VLPs, was on its own able to prevent tumor progression of growing tumors. Beyond that, in combination with an anti-PD-1 antibody treatment, the vaccine proved its full potential by eradicating tumors with a 100% efficacy. In one experiment, Ad5-MelARV alone prevented tumor growth in all vaccinated mice (Figure 6) even without co-administration of anti-PD1. However, this observation is likely due to an overall less efficient tumor growth in the challenged mice, apparent from comparing the negative controls in Figures 5 and 6. In the control group of the latter, tumors grew slower and one mouse did not develop a tumor at all. Thus, the finding of a synergistic effect between the vaccine and anti-PD-1, obtained in the experiment of Figure 5, is not annulled.

The detected immune responses induced by the adenoviral vaccine vector Ad5-MelARV were predominantly of cellular rather than humoral nature. The slight increase of antibodies directed against CT26 tumor cells is unlikely the explanation for the strong effect on target cancer cells, as serum levels were barely elevated compared to the negative control mice. This observation of insufficient antibody responses was rather surprising as the same vaccine strategy targeting human immunodeficiency virus Env [33–35] or malaria associated [36] antigens yielded markedly increased antibody levels. Furthermore, the rationale behind the vaccine strategy was that MelARV Env on the surface of released VLPs helps to cross-link B cell receptors and is presented as an exogenous antigen on major histocompatibility complex (MHC) class II molecules to CD4^+^ T cells [39, 40]. The failure of the vaccine to induce antibody responses could be related to incomplete break of immune tolerance [41], the choice of a retroviral glycoprotein which often exhibits delayed neutralization [21, 27], or the small but relevant diversity [42] between MelARV derived from C57BL/6 mice [15] and the related murine leukemia virus present in BALB/c mice [14].

As for the cellular immune responses, the increased level of activated CD8^+^ T cells specific for CT26 cells and the MelARV Env epitope AH1 [14] can potentially singularly explain the observed tumor rejection. This was not only shown by the cellular *ex vivo* assays but also the *in vivo* experiment of CD8^+^ depletion in challenged and vaccinated mice. While CD4^+^ T cell depletion had only a minor affect and could represent the lack of a promoting T helper cell activity, depletion of CD8^+^ T cells completely abrogated the protective effect of the vaccine. This shows that protection is primarily mediated by mechanisms including CD8^+^ T cells. Thus, intracellular antigens or cross-presented extracellular antigens encoded by Ad5-MelARV are likely presented on MHC class I molecules and caused the expansion of AH1-specific CD8^+^ T cells specific for the tumor. Indeed, the previously tested virus-like vaccine designs were also able to induce dramatic CD8^+^ T cell responses when adenoviruses were used as primers for Modified Vaccinia Ankara vaccines [35].

Considering that both antibody as well as T cell responses against MelARV Env are beneficial for the prevention of CT26 tumor growth [11, 25–28] the current Ad5-MelARV vaccine tested in this study could be improved by inducing higher antibody levels. Nevertheless, the T cell responses alone had an astonishing effect on the tumor rejection, even in a setting of post-challenge vaccination. The anti-tumor effects were strong enough to eradicate growing tumors efficiently when Ad5-MelARV was administered in a single vaccination. A general disadvantage of a T cell based vaccine that primarily applies to tumors in later stages is the immunosuppression by the tumor itself. In fact, also the current cancer target protein MelARV Env has immunosuppressive properties, which could help the cancer cells to escape from immunosurveillance [21–23]. This makes MelARV Env even more interesting as a tumor target. As the cancer cells need to preserve the immunosuppressive function of the MelARV Env, a down regulation of this target protein is less likely. Thus, to boost the effect of the vaccine alone, one could benefit from additionally targeting factors of the tumor escape mechanism, including the expression of T cell inactivating proteins, such as PD-1/PD-L1 or CTLA-4 [43]. The impact of the former was shown in this study by the co-administration of the T cell checkpoint inhibitor anti-PD-1. This treatment added synergistically to the Ad5-MelARV vaccination, leading to a complete eradication of existing tumors in mice. Anti-PD-1 was chosen for this treatment as it partly inhibits tumor growth of CT26 cells without preventing tumor progression completely [44], as also confirmed in this study. Anti-PD-1 is a FDA approved treatment for different types of cancer [45] and studies revealed benefits of co-administering this checkpoint inhibitor with other vaccination strategies [46]. Thus, a combinational therapy with the novel virus-like-vaccine is a realistic possibility.

The observation that Ad5-MelARV showed a high efficacy even in a post-challenge setting makes the vaccine yet more relevant for a clinical approach in which cancer is usually treated therapeutically instead of prophylactically. Since Ad5-MelARV was injected only two days after the challenge with CT26 cells before tumors were palpable, the vaccination might be seen as non-therapeutic, but this is mainly a temporal limitation of rapidly growing transplantable tumor models. Indeed, all animals did develop palpable tumors at the beginning that were rejected subsequently in most mice due to the vaccination, indicating a therapeutic-like effect of Ad5-MelARV. The subcutaneous injection of CT26 cells in BALB/c mice is a rather aggressive model system leading to the death of mice as defined by the humane endpoint within 21 days, which is not comparable to naturally occurring tumors in humans. As the immune responses take several days to develop, post-challenge vaccinations on later time points would hardly yield positive results in this murine tumor model. Especially the immunosuppressive environment developed by the growing tumors inhibits infiltration and activity of vaccine-induced cytotoxic T cells. In this regard, co-administration of Ad5-MelARV and anti-PD-1 at later tumor stages could be of interest and has to be further investigated.

One of the more surprising, yet undesired findings in this study was the observation that two intramuscular injections of a DNA vaccine yielded next to no immune responses and no benefits as a primer for an adenoviral vaccine. This seems curious in the background that the DNA vaccine evidently produce the target protein construct *in vitro*. In comparison to the adenoviral vaccine, the result could be ascribable to the simple fact that the adenovirus is more efficient in transducing target cells and thus releasing the target protein *in vivo*. Also immunogenicity of the vector likely plays a role. In this regard, xenogeneic antigens from the adenovirus capsid or even the producer cell line HEK293 might be present in the adenoviral vaccine formulation but not in the DNA vaccine and could serve as an adjuvant. However, during the purification steps via cesium chloride ultracentrifugation and gel filtration chromatography, cell-derived impurities are reduced to a minimum and should play a minor role [47]. It would be more likely that activation of innate immune pathways by the adenoviral vector, or adenovirus capsid T helper epitopes promote the observed cellular immune responses towards the encoded target protein [48]. This would resemble the previously demonstrated fusion of gp70 fragments to helper epitopes delivered as DNA vaccines [49]. Nevertheless, we have previously observed positive effects with DNA prime plus adenovirus boost and previous attempts to target murine leukemia virus sequences have achieved at least prophylactic effects with a DNA construct [30, 49]. One of the potential key differences may be that Takeda *et al*. only used the gp70 fragment of the *env* gene and needed a fusion to a genetic carrier protein to obtain responses. In our experiment we used two host proteins (Gag and Env) expressed in full length, and provided non-host antigens only in the adenoviral vector capsids. In support of the need for carrier proteins, another approach was attempted by Kershaw *et al.* where Vaccinia virus was used as a vaccine vector to encode the gp70 subunit that yielded prophylactic, but not therapeutic efficacy, unless peptide pulsed and CD40 ligand treated dendritic cell cultures were prepared *ex vivo* [29]. The fact that previous successes have been made with the gp70 alone or with smaller peptides could also imply some importance of the p15E and the imbedded immunosuppressive domain which is likely to be functional in MelARV expressing cells [21, 23]. Notably, we achieved better effects than in any of these studies by using the adenoviral vectors and encoding the full-length antigens in a VLP design, thus showing that immunosuppressive effects can be overcome by presenting the antigen in an immunogenic context. Looking forward, it may be more important if our prime-boost regimen could be improved to boost and extend immunogenicity even further. A logical approach would be to incorporate some of the previously validated techniques for making DNA vaccines immunogenic against murine leukemia viruses [49] or by using the adenovirus as a primer for a heterologous booster immunization.

A clear finding of our study was the strong T cell response obtained by Ad5-MelARV, which was necessary to eradicate growing tumors. Whether this result was mechanistically due to an efficient adenoviral introduction of the whole Env protein *in vivo* or actually the display on VLPs in a natural conformation, needs to be investigated in future experiments. Nevertheless, setting our vaccination approach into the context of previous studies that targeted MelARV/MuLV Env in CT26 tumors, there seems to be a benefit of using either adenovirus, the novel approach of encoding VLPs or both. None of the previous vaccination strategies comprising for example a gp70-encoding DNA vector in combination with adjuvants [30, 49], a recombinant Vaccinia virus encoding AH1 [29] or a peptide vaccination strategy [31], were able to clear the mice from CT26 tumors completely. In the s.c. tumor models only 20–50% of mice showed tumor free survival after prophylactic vaccinations [30, 31]. Leaving our post-challenge vaccination and the combinational therapy with anti-PD-1 aside, this is a tendency we also observed to some extent, especially in the prophylactic vaccination where 3 out of 7 mice showed a delayed tumor growth. This could be an effect of target-protein down regulation in the tumor causing an escape from immune surveillance, or the establishment of an immunosuppressive environment over time. The slightly improved protection in post-challenge settings might be explained by a general boosting of innate immune responses due to the adenoviral vector that could have an immediate effect on tumor-specific immune responses [48].

Additional to acute immune responses, immune memory and functional breadth of the vaccine was shown by a rechallenge of mice that survived a subcutaneous challenge with CT26 cells. The rechallenge was conducted with the breast cancer cell line 4T1 that expresses a variant of the MuLV Env protein [16]. Although, we did not observe 4T1-specific CD8^+^ T cells in vaccinated mice, none of the rechallenged mice developed tumors. The lack of 4T1-specific responses despite a clear presence of AH1-specific CD8^+^ T cells has been observed before in a different study and might be explained by an absence of the target protein in *in vitro* cultures of 4T1 cells [50]. Our positive result was attenuated slightly by the observation that 2 of the 7 challenged, unvaccinated control mice did not show any sign of tumor growth. This could be a consequence of immune responses toward the luciferase expressed by the tumor cells and required for tumor detection *in vivo* [51]. However, since previous studies reported only a reduction in primary tumor growth and not a complete suppression, it is possible that in these two mice luciferase-specific immune responses were combined with a reduced viability of injected tumor cells. Nevertheless, the obtained result that none of the 20 vaccinated mice developed tumors upon 4T1 cell injection showed either that protection by the administered vaccine is not restricted to only one type of cancer or, alternatively, that the first tumor has promoted cross-reactive epitope spreading. Even more convincing in this respect is the fact that the colon carcinoma cell line CT26 and the breast cancer cell line 4T1 originate from - and were implanted in - very different tissues and are therefore rather distinct from each other. Despite the different morphologies, the vaccine Ad5-MelARV was able to successfully target both types of cancer.

In conclusion, the novel vaccine strategy to target endogenous retroviruses in cancer which was tested in a murine model system showed promising results in regard to anti-tumor efficacy. As the outstanding reduction of even growing tumors was primarily attributed to cellular CD8^+^ T cells responses, further improvement is required to also address the induction of specific antibody responses. If both the humoral and cellular immunity can be triggered by our approach in murine model systems but also in human patients, this vaccine strategy of virus-encoded VLPs displaying ERV target proteins would stand out among current experimental treatment modalities and might become a valuable tool in the treatment of several ERV expressing tumors.

## MATERIALS AND METHODS

### Mice

Female BALB/c mice at 6-8 weeks of age were obtained from Envigo (Scandinavia). The mice were allowed to acclimatize for one week prior to the initiation of an experiment. All experiments were performed according to national guidelines and experimental protocols approved by the national animal experiments inspectorate (Dyreforsøgstilsynet).

### DNA and adenoviral vaccine vectors

Melanoma associated retrovirus (MelARV) *gag* and MelARV *env* were encoded in the same expression cassette, linked via a self-cleavable porcine teschovirus-1 2A peptide (P2A). The target genes were preceded by a strong cytomegalovirus promoter and a tetracycline operator site and were followed by a simian virus 40 polyadenylation signal. The expression cassette was cloned into a vaccination DNA plasmid (DNA-MelARV) or into the backbone of a replication-deficient E1 and E3 deleted human adenovirus type 5 (Ad5) vector (Ad5-MelARV).

Recombinant Ad5 vectors were produced in HEK293 cells expressing the tetracycline operator (T-REx™-293 cell line) (Thermo Fischer) [52] and an additional shRNA to further suppress target protein expression during virus production. Adenoviral particles were purified from the producer cells by cesium chloride gradient ultracentrifugation as described elsewhere [53].

In order to confirm correct insertion of the target genes, the genomes of the recombinant adenoviruses were isolated, analyzed by restriction enzyme digestion and gene sequencing.

The infectious titers of the purified viruses were determined based on the Adeno-X RapidTiter system (#632250; Clontech).

### Vaccine characterization

Vero cells were infected with 50 infectious units (IFU) of Ad5-MelARV per cell or a control Ad5 vector encoding for an irrelevant gene. Cell culture medium was changed 5 h after infection and cells were incubated in serum-free medium for 48h.

Target protein expression on the cell surface of infected cells was analyzed by flow cytometry using monoclonal antibodies against MelARV p15E (19F8; 20× concentrated cell culture supernatant from hybridomas, provided by George Cianciolo, Duke University Medical Center) or MelARV gp70 (MM2-9B6; 20× concentrated cell culture supernatant from hybridomas, provided by Tsuyoshi Takami, University of Arizona Health Sciences Center). Briefly, transduced Vero cells were incubated for 20 min with primary antibody at a dilution of 1:50 in PBS + 1% bovine serum albumin (BSA) + 0.1% NaN_3_. Target-bound antibodies were detected with an APC-labeled secondary antibody against mouse Immunoglobulin G (goat anti-mouse IgG_APC; #405308, Biolegend; diluted 1:100) in a BD LSR II Flow Cytometer.

For further analyses of target protein expression, Ad5-MelARV transduced Vero cells, or cells transduced with an irrelevant Ad5, were lysed for 30 min in NP40 lysis buffer (#FNN0021; Invitrogen) containing 7 μL/mL protease inhibitor (#P8340; Sigma-Aldrich). Additionally, vaccine-induced VLPs were purified from supernatants of transduced cells by filtration through a 0.45 μM membrane and pelleting through a 20% sucrose cushion at 82.700 g in a Beckman Coulter Ti 70 rotor using open 32 mL thickwall tubes (#355631; Beckman Coulter). After centrifugation, supernatant was removed and the VLP pellet was resuspended in PBS at 160x the original concentration. Cell lysates and purified VLPs were analyzed by Western blot for the presence of MelARV Gag and MelARV Env. To this end, 5 μg of cell lysate proteins and 2 μg of VLPs were mixed with dithiothreitol-containing Laemmli sample buffer. MelARV Gag was detected using an anti-P2A antibody (#ABS31; Millipore) at a dilution of 1:1000, while presence of the MelARV Env subunit gp70 was analyzed with the monoclonal antibody MM2-9B6 at a dilution of 1:200. Bound anti-P2A and MM2-9B6 were visualized with anti-rabbit Ig-HRP (#P0448, Dako) and anti-mouse Ig-HRP (#P0447, Dako), respectively, using LumiGLO Reserve Chemiluminescent Substrate (#54-61-00 or #54-71-02, respectively).

DNA-MelARV was analyzed regarding expression of the target construct. HEK293 cells were transduced with DNA-MelARV or DNA-SIV (negative control encoding SIV Gag + Env) using polyethylenimine. Cells were lysed as described above and analyzed by Western blot using anti-P2A to detect Gag expression.

### Immunization

Mice were vaccinated with both DNA-MelARV and Ad5-MelARV in a prime-boost regimen or with either vaccine alone. DNA was administered at a concentration of 50 μg in 50 μL TRIS/PBS (142 mM) injected intramuscularly (i.m.) in the quadriceps muscle of the right leg. Ad5 immunizations were performed subcutaneously (s.c.) in the foot pad of the right leg with 2 × 10^8^ IFU in 30 μL PBS. Control mice were injected with PBS only. For prophylactic vaccinations, mice received two DNA injections on day 1 and 22 and one Ad5 injection on day 50 or either vaccine alone on the respective dates. Mice were subsequently challenged with CT26 tumor cells on day 78. In post-challenge vaccination settings, a single dose of Ad5 was administered on day 2 after the tumor challenge. Additionally, a post-challenge vaccinated group further received a treatment of anti-PD-1 (RMP1-14; #BE0146; BioXCell) with four intraperitoneal (i.p.) injections of 200 μg antibody in 200 μL PBS on days 8, 12, 16 and 20 after the tumor challenge. A control group received only anti-PD-1 antibodies at the respective dates without Ad5 vaccination.

For CD4^+^ and CD8^+^ T cell depletion studies, mice were challenged s.c. with CT26 tumor cells on day 0 and were vaccinated with Ad5-MelARV two days later as described above. T cell depletion was performed by i.p. injection of anti-mouse CD4 (GK1.5; #BE0003-1; BioXCell), anti-mouse CD8 (2.43; #BE0061; BioXCell) or rat IgG2b isotype control (LTF-2; #BE0090; BioXCell). Antibodies were administered on days 5, 8 and 11 with 0.25 mg, 0.1 mg and 0.1 mg per mouse, respectively.

### Humoral immune responses

Blood samples of vaccinated mice were isolated one day before tumor challenge on day 77 (27 days after Ad5 vaccination) and serum was extracted by centrifugation.

To analyze tumor-specific antibodies, CT26 cells were resuspended and incubated with blood serum diluted 1:50 in PBS. Bound serum antibodies were detected by flow cytometry using an APC-labeled secondary antibody against mouse Immunoglobulin G (goat anti-mouse IgG_APC; #405308, Biolegend; diluted 1:100) in a BD LSR II Flow Cytometer.

MelARV-specific antibodies in the blood serum were measured by ELISA. MaxiSorp flat bottom plates (Thermo Fisher) were coated overnight at 4° C with a peptide of the MelARV Env subunit p15E conjugated to BSA (2 μg/mL in PBS) (BSA-CFYADHTGLVRDSMAKLRERLSQRQKLFESQQGWFEGLFNKSP; purchased from Schafer-N, Copenhagen, Denmark). Plates were blocked with 0.05% BSA buffer containing 2.07% NaCl and 0.05% Tween-20 for 2 h at 37° C. Blood serum, diluted 1:50 in blocking buffer, was added in triplicates and incubated for 3 h at 37° C. Plates were washed three times in between steps using PBS containing 2.07% NaCl and 0.1% Tween-20. Horseradish peroxidase (HRP)-conjugated polyclonal goat anti-mouse IgG (P0477, Dako) was diluted 1:2000 in blocking buffer and incubated for 2 h at 37° C. Color reactions were developed for 8 min at RT by adding TMB PLUS2 (Kem-En-Tec Diagnostics, 4395A). The HRP enzymatic reaction was terminated by addition of 2.5 M H_2_SO_4_ and the optical density was measured at 450 nm using an ELISA plate reader (VersaMax Molecular Devices).

### Cellular immune responses

MelARV Env-specific T cells were detected by enzyme-linked immunospot (ELISpot) assay analysis of splenocytes from vaccinated mice that were euthanized on day 77 (27 days after Ad5 vaccination). 2 × 10^5^ cells/well in a polyvinylidene difluoride (PVDF) 96-well plate (MSIP S4510, Millipore) were stimulated with 1 μg/mL AH1 peptide (SPSYVYHQF) in complete RPMI for 48 h under normal cell culture conditions. Unstimulated or Concanavalin A (ConA)-stimulated (2 μg/mL) splenocytes served as negative and positive controls, respectively. The assay was performed according to the instruction manual using the Mouse IFN-γ T cell ELISpot kit (CT317-PR5, U-CyTech) and spots were counted using a CTL ImmunoSpot analyzer.

Intracellular cytokine staining (ICS) was performed on splenocytes after incubation with 1 μg/mL AH1 peptide for 5 h in complete RPMI containing 50 μM 2-mercaptoethanol and 3 μM monensin under normal cell culture conditions. Stimulated splenocytes were stained with antibodies for cell surface markers (BioLegend: anti-CD4, anti-CD8, anti-CD44, anti-B220) and antibodies for intracellular cytokines (BioLegend: anti-IFNγ, anti-TNFα) as described elsewhere [54]. The data was collected on an LSRII instrument (BD Biosciences) and analyzed using FlowJo software (Tree Star, Ashland, OR). For tumor cell stimulation of T cells, splenocytes were co-incubated for 5 h with 4T1, CT26, Renca or NIH3T3 cells in a tumor cell to splenocyte ratio of 1:80. Intracellular staining was performed as described for peptide stimulation.

### Tumor challenge

To assess tumor growth *in vivo*, 5 × 10^5^ CT26 cells in 100 μL PBS were injected s.c. into the right thigh of BALB/c mice. Tumor size was measured three times a week in length and width and the tumor volume was calculated as: length * width^2^ * 0,5236 [55]. During tumor measurements, the differently vaccinated groups were blinded to prevent biased assessment. Mice were euthanized when tumors exceeded 16 mm on any side, necrotic wounds emerged or mobility of the mice was markedly reduced.

BALB/c mice that survived a previous challenge with CT26 cells were additionally injected with 2.5 × 10^4^ 4T1-Luc cells in 100 μL PBS into the thoracic mammary fat pad, 8 weeks after the initial CT26 challenge. To visualize tumor formation 6 weeks after 4T1 challenge, mice were injected i.p. with Luciferin (1.5 mg per 10 g mouse) and tumors were imaged 12 min after injection using an IVIS Spectrum *in vivo* imaging system.

### Statistical analyses

All statistical analyses were performed using GraphPad Prism software (v5.03). Groups were compared using two-tailed, unpaired Mann-Whitney tests. Significances are indicated by asterisks: ^*^(*P* ≤ 0.05); ^**^(*P* ≤ 0.01); ^***^(*P* ≤ 0.001). When comparing different groups of vaccinated mice, results are shown as a mean of each group with standard error of mean (SEM).

## SUPPLEMENTARY MATERIALS FIGURE



## Abbreviations

Ad5: adenovirus type 5
BSA: Bovine serum albumin
DNA: deoxyribonucleic acid
ELISA: Enzyme-linked immunosorbent assay
ELISpot: Enzyme-linked immunospot assay
Env: envelope protein
ERV: Endogenous retrovirus
Gag: group-specific antigen
HERV: Human endogenous retrovirus
HRP: horseradish peroxidase
IFNγ: interferon gamma
IFU: Infectious units
i.m.: intramuscularly
i.p.: intraperitoneal
ISD: Immunosuppressive domain
Luc: luciferase
MelARV: Melanoma associated retrovirus
MHC: Major histocompatibility complex
MuLV: Murine leukemia virus
NaN_3_: Sodium azide
PBMC: Peripheral blood mononuclear cell
PBS: phosphate buffered saline
s.c.: subcutaneously
shRNA: small hairpin ribonucleic acid
SEM: Standard error of mean
TNFα: tumor necrosis factor alpha
VLP: Virus-like particles.
