# Supplementary material for: Adenovirus based virus-like-vaccines targeting endogenous retroviruses can eliminate growing colorectal cancers in mice
Source: Oncotarget. 2019 Feb 15;10(14):1458–72. doi: 10.18632/oncotarget.26680 (PMC6402721; doi:10.18632/oncotarget.26680)
Supplement: Supplementary file 1 [file oncotarget-10-1458-s001.pdf]

Adenovirus based virus-like-vaccines targeting endogenous retroviruses can eliminate growing colorectal cancers in mice

SUPPLEMENTARY MATERIALS

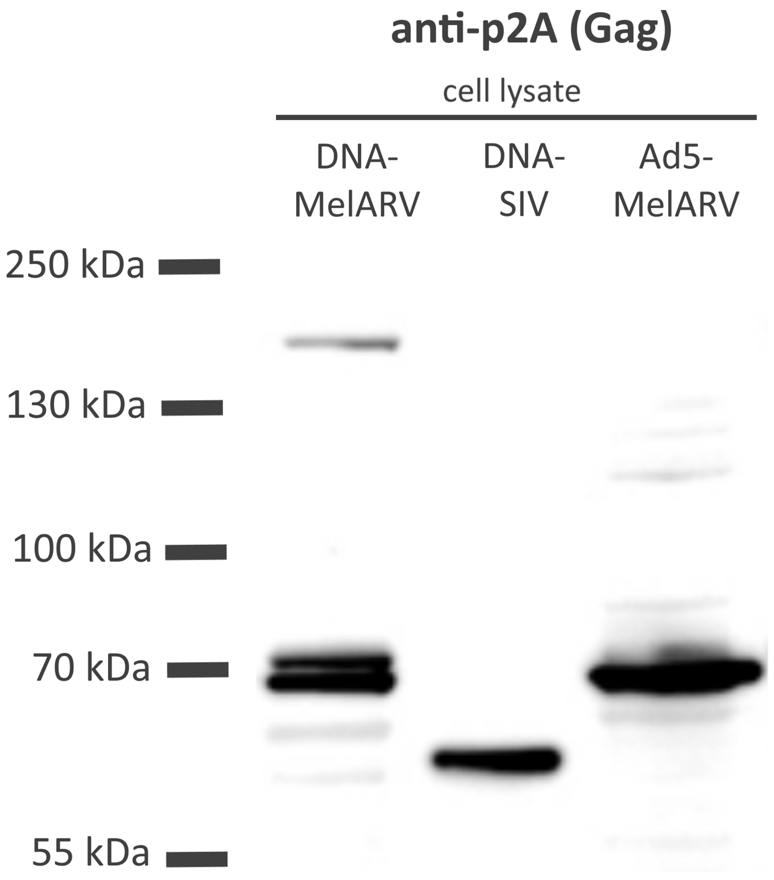

Supplementary Figure 1: Expression test of vaccine vector DNA-MelARV. Functionality of DNA-MelARV, encoding MelARV Gag and Env coupled by P2A, was shown by detecting target protein expression in HEK293 cells. Anti-P2A was used to detect Gag in lysates of DNA-MelARV transduced cells by Western blot. DNA encoding for SIV Gag and Env coupled by P2A (DNA-SIV, Ø) and Ad5-MelARV served as negative and positive controls, respectively. Next to the expected band size of ~70kDa in the DNA-MelARV sample showing Gag, the protein band of high molecular weight (~150 kDa) represents uncleaved MelARV Gag-P2A-Env protein. The observed band of ~60 kDa in the negative control represents the SIV Gag with a smaller molecular weight than MelARV Gag.
